# Guaroa Virus and *Plasmodium vivax* Co-Infections, Peruvian Amazon

**DOI:** 10.3201/eid2604.191104

**Published:** 2020-04

**Authors:** Crystyan Siles, William H. Elson, Stalin Vilcarromero, Amy C. Morrison, Robert D. Hontz, Freddy Alava, Hugo Valdivia, Vidal Felices, Carolina Guevara, Sarah Jenkins, Eugenio J. Abente, Julia S. Ampuero

**Affiliations:** US Naval Medical Research Unit 6, Lima, Peru (C. Siles, S. Vilcarromero, R.D. Hontz, H. Valdivia, V. Felices, C. Guevara, S. Jenkins, E.J. Abente, J.S. Ampuero);; University of California, Davis, Davis, California, USA (W.H. Elson, A.C. Morrison);; Centro de Salud San Juan, Direccion Regional de Salud Loreto, Iquitos, Peru (F. Alava)

**Keywords:** Guaroa virus, Orthobunyavirus, Plasmodium vivax, co-infection, Peru, parasites, viruses, vector-borne infections, Peruvian Amazon, malaria

## Abstract

During April–June 2014 in a malaria-endemic rural community close to the city of Iquitos in Peru, we detected evidence of Guaroa virus (GROV) infection in 14 febrile persons, of whom 6 also had evidence of *Plasmodium vivax* malaria. Cases were discovered through a long-term febrile illness surveillance network at local participating health facilities. GROV cases were identified by using a combination of seroconversion and virus isolation, and malaria was diagnosed by thick smear and PCR. GROV mono-infections manifested as nonspecific febrile illness and were clinically indistinguishable from GROV and *P. vivax* co-infections. This cluster of cases highlights the potential for GROV transmission in the rural Peruvian Amazon, particularly in areas where malaria is endemic. Further study of similar areas of the Amazon may provide insights into the extent of GROV transmission in the Amazon basin.

Since 1990 in Peru, the US Naval Medical Research Unit 6 (NAMRU-6), in collaboration with the Peruvian Ministry of Health, has conducted clinic-based passive surveillance of acute febrile illnesses in Iquitos, the largest city in the Peruvian Amazon. Iquitos is an urban locale in the heart of the Amazon rainforest; the climate is tropical with frequent heavy rainfall. It has a population of ≈400,000, is accessible only by river or air travel, and is situated 120 m above sea level at the confluence of the Nanay, Itaya, and Amazon Rivers in the Loreto Department of northeastern Peru. Iquitos comprises 4 districts: Iquitos, San Juan, Belen, and Punchana. The city and its surrounding periurban and rural areas are home to an abundance of mosquito species and provide a suitable environment for arbovirus and *Plasmodium* spp. transmission. Since 1993, at least 13 arboviruses have been detected in this area, of which dengue virus (DENV), Zika virus, Mayaro virus, and Venezuelan equine encephalitis virus are considered to be of public health importance (*1*).

Guaroa virus (GROV; order *Bunyavirales*, family *Peribunyaviridae*, genus *Orthobunyavirus*) (*2*) is a known cause of febrile illness in tropical regions of Central and South America (*3*). It was first isolated from asymptomatic humans in Colombia in 1956 (*4*) and isolated from symptomatic humans in Brazil in 1964 (*5*). Recent phylogenetic analysis suggests that a common ancestor of GROV and Wyeomyia virus was introduced into South America in the Brazilian Amazon region ≈250 years ago, with subsequent southward spread of GROV to Peru within the past 60–70 years (*6*). A serologic survey in 1965 provided evidence of GROV transmission in Peru shortly after this time (*7*).

More recent antibody prevalence studies on samples collected in Iquitos in 2006 demonstrated an overall GROV seroprevalence of 13% (*3*). The increasing seroprevalence with age suggests that transmission occurred consistently in the region over several years (*3*). In addition to seroprevalence, this study also described 15 symptomatic GROV infections in Peru (including 3 in Iquitos) during 1995–2008, providing further evidence of GROV as a cause of symptomatic disease in the region (*3*).

The only confirmed vector of GROV is *Anopheles* (*Kerteszia*) *neivai* mosquitoes (*8*). This species is an important vector of human malaria in the Pacific lowlands of Colombia (*9*). After a successful elimination effort in the 1960s, malaria reemerged in the early 1990s in the Peruvian Amazon, coincident with the reintroduction of *An. darlingi* mosquitoes and is currently a leading cause of febrile illness (*10*). Subsequently, a resurgence of *P. falciparium* peaked in 1997, at which point *P. falciparium* prevalence decreased significantly. The *An. darlingi* mosquito is the primary vector of malaria in rural areas near Iquitos (*11*–*13*). There are numerous reports in the literature of arboviral and malarial co-infections, but reports of GROV and malaria co-infection are scarce, and only co-infection with *P. falciparium* has been reported (*14*,*15*). We describe the clinical and epidemiologic aspects of a GROV outbreak in the rural surroundings of Iquitos and report symptomatic co-infections with GROV and *P. vivax*.

## Methods

We identified GROV cases through a passive febrile surveillance system at 12 health centers in urban, periurban, and rural areas in and around Iquitos, which were described previously (*1*). Inclusion criteria were age >5 years, oral or tympanic temperature >38°C (or axillary >37.5°C), duration of symptoms <5 days, and no obvious focus of infection. All participants at these sites were initially screened for *Plasmodium* infection by thick smear and were then invited to undergo screening for arboviruses regardless of their smear results. In addition to serum samples obtained during the acute phase, serum samples were obtained during follow-up evaluations 20 days (± 10 days), 3 months (± 15 days), 6 months (± 15 days), and 12 months (± 30 days) after the initial sample. Serum samples collected on day 20 were used to measure convalescent titers. 

Of the 12 clinics, 2 are in rural communities with active malaria transmission (Zungarococha and Quistococha communinities), and 3 urban clinics serve as catchment areas for communities with active malaria transmission (Bella Vista Nanay, 6 de Octubre, and San Juan). The GROV cases reported in this study were captured during April 5–June 26, 2014, from 3 malaria-endemic communities in the district of San Juan (Santo Tomas, Quistococha, and Zungarococha) (Figure).

**Figure:**
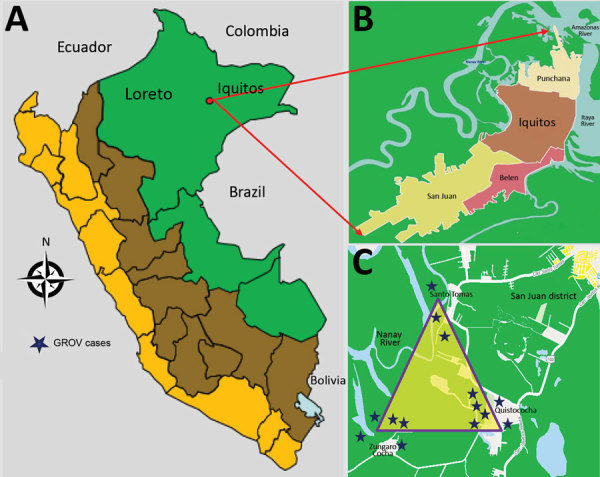
Geographic distribution of patients with Guaroa virus infection, April–June 2014. A) Peru. B) Iquitos districts. C) District of San Juan. Stars indicate locations of Guaroa virus cases.

We obtained approval for the study protocol (NMRCD.2010.0010) from the Institutional Review Board of NAMRU-6 in compliance with all applicable US federal regulations governing the protection of human subjects and from the Institutional Review Board of the Peruvian Ministry of Health. Adults >18 years of age provided written informed consent, and parents or legal guardians of participants <17 years of age provided assent.

### Arboviral Testing

After a negative result by reverse transcription PCR (RT-PCR) for DENV (*16*), we attempted to isolate causative agents with *Aedes albopictus* (C6/36) and African green monkey kidney (Vero 76) cell cultures in combination with an immunofluorescence assay using hyperimmune mouse ascitic fluid (HMAF) raised against flaviviruses, alphaviruses, and bunyaviruses. For bunyavirus detection, we used pooled HMAF against various bunyaviruses (Oropouche 172, Caraparu isolate from Peru, Guaroa isolate from Peru, Maguari R18134, Echarate isolate from Peru and California [EVBSF-283]), followed by the addition of fluorescein-conjugated goat antimouse IgG (*1*). We performed ELISA IgM capture assay with acute and convalescent serum samples for GROV and other endemic arboviruses (Mayaro virus, Venezuelan equine encephalitis virus, Oropouche virus, group C viruses, and DENV). Microtiter plates (96-well format) were coated with goat F(ab′)2 antihuman IgM (Jackson Inmuno Research Laboratories Inc., https://www.jacksonimmuno.com ) diluted 1∶1,000 in phosphate-buffered saline and incubated overnight at 4°C. We diluted participant serum 1∶100 and incubated in coated wells for 1 h at 37°C, then added viral antigen and incubated at 37°C for 1 h. We detected viral antigens with HMAF (produced by inoculation of mice with the respective viral strains), followed by horseradish peroxidase–conjugated goat antimouse IgM + IgG (H+L) (Thermo Fisher Scientific, https://www.thermofisher.com). After adding ABTS (2,2¢-azino-bis-[3-ethylbenzthiazoline-6-sulfonic acid]) colorimetric substrate, we read plates at 410 nm with a MultisKan Microplate Photometer FC absorbance reader (Thermofisher Scientific) (*1*). We retested samples with detectable IgM at 4-fold serial dilutions (1:100, 1:400, 1:1,600, and 1:6,400). We defined seroconversion as a >4-fold increase in IgM between acute- and convalescent-phase samples and considered participants positive for GROV infection if virus was isolated by cell culture or if seroconversion was observed.

### RNA Extraction, Sequencing, and Phylogenetic Analysis

We extracted RNA from 14 serum samples using the QIAamp Viral RNA Mini Kit (QIAGEN, https://www.qiagen.com), following the manufacturer’s instructions. We performed reverse transcription and amplifications using the Access RT-PCR System kit (Promega) and previously described primers Bunya 1 (GTCACAGTAGTGTACTCCAC) and Bunya 2 (CTGACAGTAGTGTGCTCCAC), which amplify a 616-bp amplicon of the S (small) RNA segment. The amplicon covers 462/702 nt of the nucleocapsid coding sequence and 154/226 nt of the 5′-nontranslated region of the viral RNA. We performed reverse transcription at 42°C for 1 h. PCR amplifications comprised 38 cycles of denaturation (94°C for 30 s), annealing (50°C for 40 s), and extension (72°C for 1.5 min) and a final extension at 72°C for 10 min. We then purified amplicons with Centri-Sep columns (Invitrogen, https://www.thermofisher.com) and sequenced directly using the BigDye Terminator v3.1 Sequencing Kit (Applied Biosystems, https://www.fishersci.com), following the manufacturer’s protocol. We conducted sequencing on a 3130 XL Genetic Analyzer (Applied Biosystems) platform and analyzed sequences using Sequencher software (Gene Codes Corporation); we queried individual sequences by using the nucleotide database with BLAST (*17*).

### Malaria Testing

Microscopy was the primary method of *Plasmodium* identification conducted by the health centers as part of the Ministry of Health’s surveillance. Microscopy was also used as part of our study, although to evaluate *Plasmodium* spp. infection more accurately in all GROV-positive cases, we also tested blood from acute-phase samples for *Plasmodium* by PCR regardless of their initial thick-smear results. DNA was extracted from whole blood samples using the DNeasy Blood & Tissue Kit (QIAGEN), following the manufacturer’s protocol. *Plasmodium* DNA was amplified using a nested PCR protocol that targets the small subunit ribosomal RNA 18S gene (*18*). Both reactions were conducted in a volume of 50 μL containing 1X Taq polymerase buffer (Invitrogen), 2 mmol/L MgCl2, 125 μmol/L dNTPs (Invitrogen), 0.25 μmol/L of each primer, 1 unit of Taq DNA polymerase (Invitrogen), and 5 μL of DNA sample. We ran both PCR reactions on a Verity Thermal Cycler (Applied Biosystems) as previously described (*18*). We used DNA from *P. falciparum* 3D7 and *P. vivax* Sal-I reference strains as positive controls and human DNA from a person from a non–malaria-endemic area as a negative control. We subsequently ran PCR products on a 2% agarose gel; a band of ≈205-bp indicates the presence of *P. falciparum* DNA, and a PCR product of 120-bp indicates the presence of *P. vivax* DNA.

## Results

During the 3-month period when GROV infections were detected, a total of 681 febrile patients were enrolled from all 12 participating clinics around Iquitos. All GROV-infected participants lived in the district of San Juan, in 1 of the 3 malaria-endemic communities: Santo Tomas, Quistococha, or Zungarococha. A total of 121 (18%) of 681 febrile persons resided in 1 of these 3 communities in San Juan. Of those, 14 (12%) tested positive for GROV infection, of whom 6 (43%) were co-infected with *P. vivax*. Of the 14 persons for whom GROV infection was confirmed, 3 were enrolled in urban health facilities, although these 3 persons reside in the rural San Juan district (Figure). During the 3-month study period, 26 malaria cases were reported in the San Juan district, including the 6 with GROV co-infection.

All 14 GROV-infected persons seroconverted and were negative for all other examined arboviruses; 11 (79%) samples collected from these persons yielded GROV isolates in both Vero-76 and C6/36 (Table 1). Diagnosis of malaria in 6 GROV-infected persons was determined by positive thick smear in 4 cases, positive PCR and thick smear in 1 case, and positive PCR alone in 1 case.

**Table 1:** Laboratory findings and serologic response expressed as inverse titers for patients with GROV infection, Peruvian Amazon, April–June 2014*

Patient	Thick smear malaria result			Virus isolated	IgM				
		PCR result			Acute phase	Convalescent phase†	Month 3	Month 6	Month 12
		Malaria	GROV						
1	*Plasmodium vivax*	Neg	Pos	GROV	0	1:6,400	0	0	0
2	*P. vivax*	Neg	Pos	GROV	0	0	1:1,600	0	0
3	Neg	Neg	Neg	Neg	1:400	1:1,600	0	VNC	VNC
4	Neg	Neg	Pos	GROV	0	1:1,600	0	0	0
5	Neg	Neg	Pos	GROV	0	1:1,600	0	0	0
6	*P. vivax*	Neg	Pos	GROV	0	1:6,400	0	0	0
7	*P. vivax*	*P. vivax*	Pos	GROV	0	1:6,400	1:400	1:100	0
8	Neg	Neg	Pos	GROV	0	1:6,400	0	0	0
9	*P. vivax*	Neg	Pos	GROV	0	1:6,400	0	0	0
10	Neg	Neg	Neg	GROV	0	1:6,400	0	0	0
11	Neg	*P. vivax*	Neg	Neg	1:100	1:1,600	VNC	VNC	VNC
12	Neg	Neg	Pos	GROV	0	1:400	0	0	0
13	Neg	Neg	Neg	Neg	0	1:1,600	VNC	VNC	VNC
14	Neg	Neg	Pos	GROV	0	1:6,400	1:400	0	0

*GROV, Guaroa virus; neg, negative; pos, positive; VNR, visit not complete. †At follow-up 20 d after the acute phase.

Mean age of the 14 GROV-infected persons was 35.4 years (range 14–64 years). The mean age of the 8 GROV mono-infected persons was 40 years (range 40–64 years), and the mean age of co-infected persons was 29.2 years (range 17–35 years). Nine of the 14 GROV-positive case-patients were male.

Ten samples were positive for GROV by RT-PCR and were sequenced. A BLAST search determined that the DNA sequences had 99% identity with 3 GROV strain isolates collected in Peru during 2004–2008. Multiple alignment analysis of the 10 sequences analyzed showed they were nearly identical, with only 2 nt differences: a synonymous substitution at codon 151 of the nucleocapsid (TTT and TTC) and in the 5′-nontranslated region of the viral RNA at nucleotide position 31 (C and T; Appendix Figure).

All participants reported fever, chills, malaise, body pain, joint pain, and headache (Table 2). Myalgia, anorexia, nausea, and dysgeusia occurred in 8 patients. Only 2 patients reported a rash. Distinct symptom frequencies between persons with GROV mono-infection and co-infected persons were conjunctival injection, dysgeusia, cough, and sore throat. These symptoms occurred more frequently in persons without malaria. These differences in symptoms were not statistically significant (χ^2^ with Yates correction significance level 0.05).

**Table 2:** Clinical, epidemiologic, and demographic characteristics of patients confirmed to have GROV mono-infection and GROV–*Plasmodium* sp. co-infection, Amazonian Peru, April–June 2014*

Patient no.	Age, y/sex	Occupation	Day of illness at enrollment	Infection	Acute-phase clinical manifestations
1	40/F	Housewife	5	Co-infection	Constitutional (malaise, chills, retro-orbital pain, dizziness,headache), gastrointestinal (anorexia, dysgeusia, nausea), musculoskeletal (myalgia, bone pain, joint pain)
2	53/F	Housewife	4	Co-infection	Constitutional (malaise, chills, retro-orbital pain, conjunctivitis, headache), gastrointestinal (anorexia, nausea, abdominal pain), musculoskeletal (myalgia, bone pain, joint pain)
3	64/M	Farmer	3	GROV	Constitutional (malaise, chills, dizziness, retro-orbital pain, conjunctivitis, headache), gastrointestinal (anorexia, dysgeusia, abdominal pain, nausea), musculoskeletal (myalgia, bone pain, joint pain), cutaneous (exanthema)
4	38/M	Farmer	1	GROV	Constitutional (malaise, chills, dizziness, retro-orbital pain, conjunctivitis, headache), gastrointestinal (anorexia, dysgeusia, nausea), musculoskeletal (myalgia, bone pain, joint pain), respiratory (rhinorrhea, sore throat, cough)
5	51/F	Housewife	1	GROV	Constitutional (malaise, chills, retro-orbital pain, conjunctivitis, headache), gastrointestinal (anorexia, dysgeusia, diarrhea, abdominal pain), musculoskeletal (myalgia, bone pain, joint pain)
6	25/F	Housewife	2	Co-infection	Constitutional (malaise, chills, dizziness, retro-orbital pain, conjunctivitis, headache), gastrointestinal (anorexia, dysgeusia, abdominal pain, diarrhea, nausea), musculoskeletal (myalgia, bone pain, joint pain), cutaneous (exanthema), hemorrhagic (gingivorrhagia)
7	22/F	Housewife	2	Co-infection	Constitutional (malaise, chills, retro-orbital pain, headache), gastrointestinal (anorexia, dysgeusia, nausea), musculoskeletal (myalgia, bone pain, joint pain)
8	36/M	Farmer	2	GROV	Constitutional (malaise, chills, dizziness, retro-orbital pain, conjunctivitis, headache), gastrointestinal (anorexia, dysgeusia, nausea), musculoskeletal (myalgia, bone pain, joint pain), respiratory (rhinorrhea)
9	18/M	Army forces	1	Co-infection	Constitutional (malaise, chills, dizziness, retro-orbital pain, headache), gastrointestinal (anorexia, dysgeusia, abdominal pain), musculoskeletal (bone pain, joint pain)
10	29/M	Army forces	4	GROV	Constitutional (malaise, chills, dizziness, headache), gastrointestinal (dysgeusia, abdominal pain, diarrhea), musculoskeletal (myalgia, bone pain, joint pain)
11	17/M	Student	3	Co-infection	Constitutional (malaise, chills, dizziness, headache), gastrointestinal (anorexia, nausea), musculoskeletal (myalgia, bone pain, joint pain)
12	24/M	Student	1	GROV	Constitutional (malaise, chills, retro-orbital pain, conjunctivitis, headache), gastrointestinal (anorexia, dysgeusia), musculoskeletal (myalgia, bone pain, joint pain)
13	64/M	Farmer	5	GROV	Constitutional (malaise, chills, dizziness, retro-orbital pain, conjunctivitis, headache), gastrointestinal (anorexia, dysgeusia, abdominal pain, vomiting, diarrhea, nausea), musculoskeletal (myalgia, bone pain, joint pain), cutaneous (exanthema)
14	14/M	Student	1	GROV	Constitutional (malaise, chills, retro-orbital pain, conjunctivitis, headache), gastrointestinal (anorexia, dysgeusia, nausea), musculoskeletal (myalgia, bone pain, joint pain), respiratory (rhinorrhea, sore throat, cough)

*GROV, Guaroa virus.

Median fever duration for both GROV mono-infection and co-infected persons was 4.5 days. Symptoms with the longest median duration for all participants were body pain, joint pain, headache, and dizziness (all >5 days). Headache was reported during the convalescent phase and for up to 3 months during follow-up in 4 patients with a GROV mono-infection and 2 patients with GROV and malaria co-infection. Duration of symptoms between mono-infected and co-infected persons did not differ significantly. No patients were hospitalized, and all recovered without sequelae.

## Discussion

The circulation of many arboviruses in the Peruvian Amazon is well documented, but most cases are not reported (*1*), possibly because the asymptomatic and mild self-limiting infections are common and do not usually result in treatment-seeking behavior (*14*). Other important factors are the absence of diagnostic facilities for detecting arboviruses in areas to which they are endemic, limited access to healthcare for at-risk persons in rural Amazonian populations, and the narrow window of opportunity to collect potentially diagnostic blood samples during the acute phase of the illness (*15*,*19*). Another factor limiting arboviral diagnosis and identification of co-infections with other pathogens is that once malaria is diagnosed, secondary diagnoses are rarely pursued (*15*). For example, in our study GROV infection was diagnosed in 3 patients after they returned to the same health center within 3 days after having tested positive for *P. vivax*. Because their febrile symptoms persisted despite treatment with antimalarial drugs, they provided additional samples that tested positive for GROV. Although previous exposure to antimalarial drugs might have affected our RT-PCR results for malaria and might have resulted in a false-negative result, only 2 patients were willing to provide blood samples for arbovirus testing during the same visit in which they tested positive for *P. vivax* by thick smear. The detection of viral and parasitic co-infection demostrates the value of actively looking for viral pathogens in malaria-positive patients at the time they seek medical care during the acute phase, when the probability of identifying a viral infection is highest.

Current knowledge about GROV as a human pathogen is limited, and little is known about its reservoir hosts and vectors, particularly in Peru. *Anopheles* mosquitoes have been implicated elsewhere as vectors for GROV (*5*,*8*,*20*), although few arbovirus are known to be transmitted by *Anopheles* mosquitoes, such as o’nyong-nyong virus, which is transmitted in Africa by *An. funestus* and *An. gambiae* mosquitoes (*21*). It is possible that a co-infected mosquito could transmit both *Plasmodium* spp. and GROV; however, vector-competence studies of *An. darlingi* mosquitoes with GROV have not yet been published (*22*). The *An. darlingi* mosquito is the most common and competent malaria vector in the Peruvian Amazon (*11*–*13*,*23*,*24*). During 2014, the Peruvian Ministry of Health recorded 65,239 malaria cases in the entire country, most of which resulted from *P. vivax* (84%) and were detected in the Amazon region of Loreto (≈90%) (*23*). The GROV infection reported in this study coincides temporally with the peak of this *P. vivax* outbreak observed in 2014 (*25*), increasing the chances of co-infected mosquitoes, probability of transmission to humans, or both.

Furthermore, malaria rates tend to be high along the Nanay River, located only a few kilometers from the southern Iquitos city limits (*26*). All the GROV cases observed in the present report came from rural communities in the district of San Juan Baustista bordering the Nanay River. Further investigation is needed to understand the nature of these co-infections because they could have occurred solely as a consequence of the elevated levels of circulating *P. vivax*, a possible peak in *Anopheles* populations, or subclinical/persistent malaria with fever caused by GROV infection. In addition, identification of nonhuman reservoirs for GROV is critical to clarify the epidemiology of the disease and distinguish GROV from other arboviruses circulating in and near Iquitos (*27*,*28*).

Consistent with a previous report (*3*), the clinical manifestations of GROV infection in this study were highly nonspecific, and persons co-infected with *P. vivax* were clinically indistinguishable from those with GROV infection alone. It was not possible to determine the relative contribution of malaria and GROV to the symptomatology of co-infected persons; however, 3 of the 5 patients with positive thick smears who were treated with antimalarial drugs returned to the same health center within 3 days after the initial diagnosis because of persistent fever. These symptoms could, at least in part, have been related to the GROV infection consistent with other clinical symptom reports (*3*). Understanding the origin of symptoms in co-infected persons is further complicated because both asymptomatic malaria and arboviral disease are common (*14*,*29*).

The cases of GROV fever we report may underrepresent the true number of total cases in Iquitos during the study period. GROV may be underreported because of overlapping symptoms between pathogens, such as DENV and *Plasmodium* spp., that are more frequently targeted by diagnostic tests. Our study highlights the importance of the febrile surveillance system and its access to advanced diagnostic facilities that enabled detection of these cases. Continued surveillance is necessary not only to monitor the dynamics of well-studied diseases, such as dengue and malaria, but also to capture the emergence of potentially new pathogens with epidemic potential that pose a public health risk. In particular, examining malaria-endemic communities may provide an opportunity to better quantify the incidence of GROV and other endemic or enzootic pathogens.

Appendix Additional results for study of Guaroa virus and *Plasmodium vivax* co-infections, Peruvian Amazon.
